# Comparison between one-stage and two-stage CT-guided localization of lung nodules with surgical resection: a single medical center experience

**DOI:** 10.1186/s13019-024-02823-7

**Published:** 2024-05-30

**Authors:** Kai-Yun Hsueh, En-Kuei Tang, Fu-Zong Wu, Ping-Chung Tsai, Chih-Wen Shu, Yen-Chiang Tseng, Yih-Gang Goan

**Affiliations:** 1 Division of Thoracic Surgery, Department of Surgery, Kaohsiung Veterans General Hospital, Kaohsiung, Taiwan; 2 School of Medicine, National Yang Ming Chiao Tung University, Taipei, Taiwan; 3 College of Medicine, Kaohsiung Medical University, Kaohsiung, Taiwan; 4 Department of Radiology, Kaohsiung Veterans General Hospital, Kaohsiung, Taiwan; 5 Institute of BioPharmaceutical Sciences, National Sun Yat-Sen University, Kaohsiung, Taiwan; 6 Department of Biomedical Science and Environmental Biology, Kaohsiung Medical University, Kaohsiung, Taiwan; 7 Institute of Clinical Medicine, National Yang Ming Chiao Tung University, Taipei, Taiwan; 8 Department of Pharmacy and Master Program, Tajen University, Pingtung, Taiwan; 9 Division of Thoracic Surgery, Department of Surgery, Pingtung Veterans General Hospital, Pingtung, Taiwan

**Keywords:** CT-guided localization, Pulmonary lesions, Minimally invasive VATS

## Abstract

**Background:**

This retrospective study aimed to compare the efficacy and safety of one-stage computed tomography (OSCT)- to that of two-stage computed tomography (TSCT)-guided localization for the surgical removal of small lung nodules.

**Methods:**

We collected data from patients with ipsilateral pulmonary nodules who underwent localization before surgical removal at Veteran General Hospital Kaohsiung between October 2017 and January 2022. The patients were divided into the OSCT and TSCT groups.

**Results:**

We found that OSCT significantly reduced the localization time and risky time compared to TSCT, and the success rate of localization and incidence of pneumothorax were similar in both groups. However, the time spent under general anesthesia was longer in the OSCT group than in the TSCT group.

**Conclusions:**

The OSCT-guided approach to localize pulmonary nodules in hybrid operation room is a safe and effective technique for the surgical removal of small lung nodules.

## Introduction

Lung cancer is the leading cause of death from cancer worldwide [1]. Low-dose chest computed tomography (CT) for surveying lung cancer is increasingly used worldwide, and many small lung nodules are detected early nowadays [2, 3]. Most of these small pulmonary nodules are suggested to be observed and regularly followed up at outpatient departments; however, some patients may be anxious about malignant tendencies and request to undergo surgery to remove these lesions. These tiny pure ground-glass nodules (GGN) in the lungs are difficult to detect manually for resection during surgery. CT-guided lesion localization is helpful for resection because the precise location of a lesion is determined using a dye or a hookwire [4], which guides the surgeon to ensure the exact location of a target lesion and makes the operation smooth and safe. Traditionally, the two-stage CT-guided localization (TSCT), also known as pre-operative CT-guided localization, is used to localize tiny pure GGN or deep GGN in the lungs. Recently, the emergence of the hybrid operation room (HOR) makes one-stage CT-guided (OSCT) localization, also known as intraoperative CT-guided localization, more convenient and easier. The advent of the HOR has allowed the simultaneous localization and removal of small lung nodules. Some studies have demonstrated more benefits of OSCT localization in terms of safety and efficacy than TSCT localization [5]. This report on a single-center experience aims to compare the efficacy and safety of TSCT to those of OSCT.

## Materials and methods

### Study design and setting

This single-center study was a retrospective study aimed to compare the efficacy and safety of TSCT to those of OSCT. In this study, we collected data from patients with ipsilateral pulmonary nodules in whom localization was performed before surgical removal between October 2017 and January 2022 at Veteran General Hospital Kaohsiung (VGHKS). Ethical review and approval were waived for this study because our data were retrospectively collected from the data base of VGHKS. We retrieved raw data from the database and tracked the follow-up conditions of the patients. The number of cases included in our study was determined to be statistically significant based on consultation with a statistics expert, and data cutoff date was on January 31, 2022. The study protocol was approved by the Ethics Committee of the Veteran General Hospital, Kaohsiung (approval number: KSVGH-CT8-07). All participants provided written informed consent.

### Study patients

**Fig. 1:**
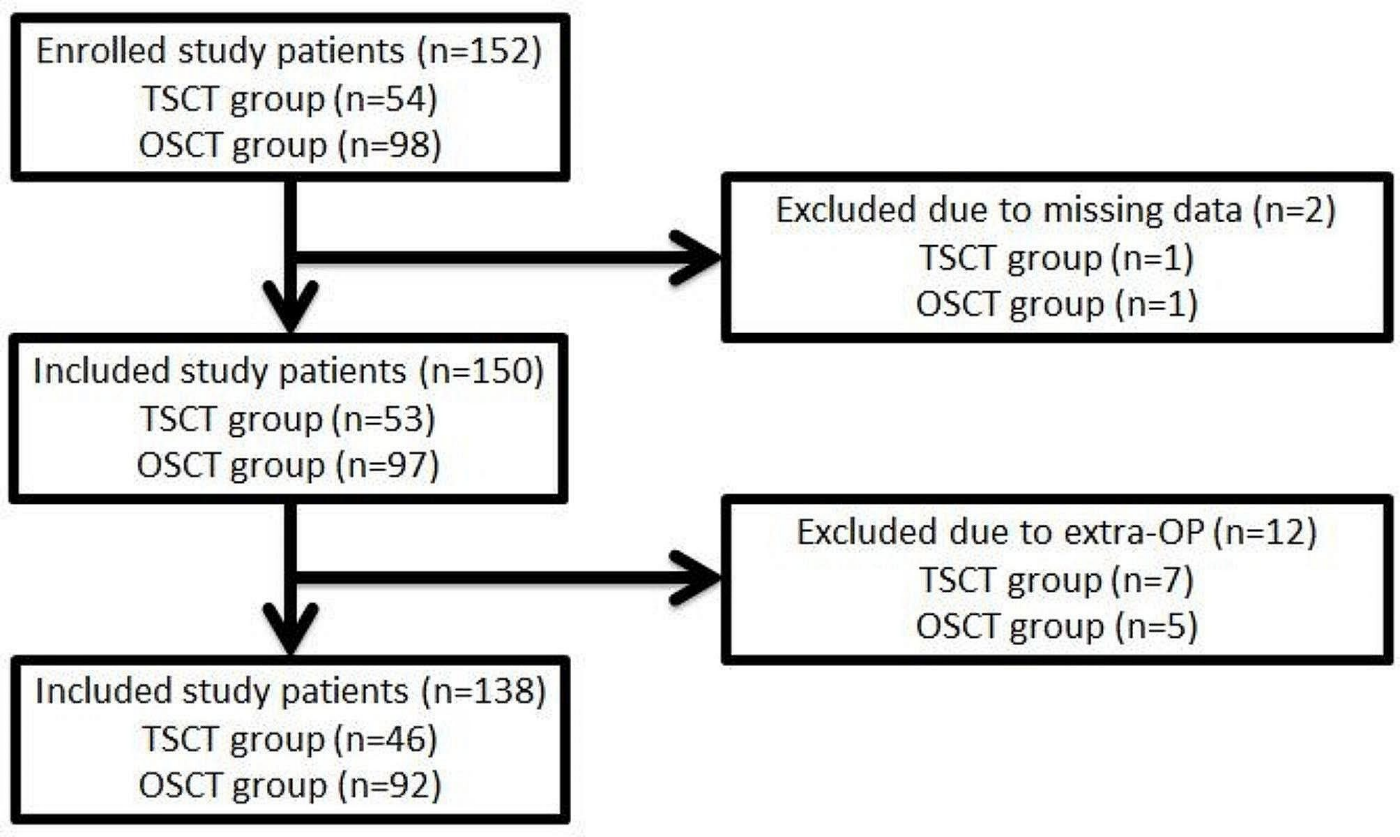
Flowchart of the patient selection process

We collected the study patients who underwent localization and removal of small lung lesions (Fig. 1). The study patients might receive OSCT-guided approach or TSCT-guided method according to the database. The indication to localizing pulmonary lesions and surgery to remove the nodules were the same in both groups. Our participants were divided into two groups; i.e., the OSCT and TSCT groups. Indications include the fact that the tiny solid nodules measured less than 10 mm [6] or deep location more than 10 mm (distance from lesion site to visceral pleura). Any subsolid nodules (pure GGN), regardless of size or depth, are considered to be localized. Before HOR has been opened in 2019, the TSCT localization method has been the standard method to approach the tiny pulmonary lesions. In 2019, our HOR was launched, and we subsequently adopted the OSCT localization approach.

### Two-stage computed tomography-guided localization

TSCT localization was performed by an experienced radiologist in the CT suite. Most of the patients were positioned in the supine position because of the limitation of space in the CT scanner. The target lesion was scanned and the location was shown on the monitor. The puncture site was sterilized carefully, and the radiologist pierced into the lung near the target lesion with a 10-cm long, 20-gauge cannula needle housing a hookwire [6]. Once the cannula needle was inserted into the target site, the hookwire was placed. After localization was completed, the patient was transferred to the original ward to wait for information about surgery. Then, the patient would be transferred to the operation room to undergo wedge resection for the removal of pulmonary lesions. Once the lesion was removed, the specimen was sent for a frozen section to check the tissue components. If the frozen section results reveal invasive adenocarcinoma, a lobectomy would be performed. If the frozen section results reveal atypical adenomatous hyperplasia (AAH), adenocarcinoma in situ (AIS), or minimally invasive adenocarcinoma (MIA), a lobectomy would not need to be performed [6].

### One-stage computed tomography-guided localization

**Fig. 2:**
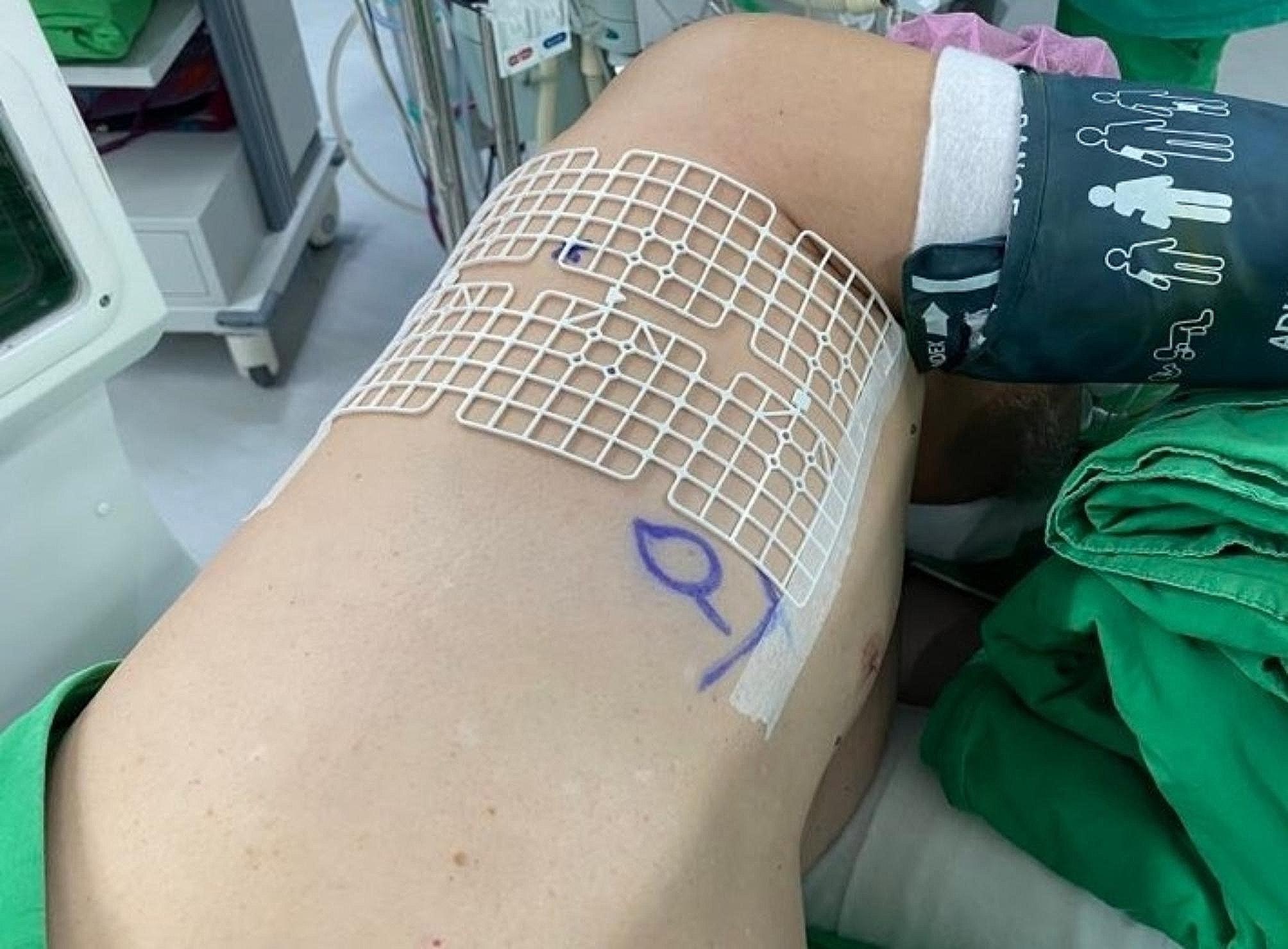
Grid placed on the chest wall for localization

**Fig. 3:**
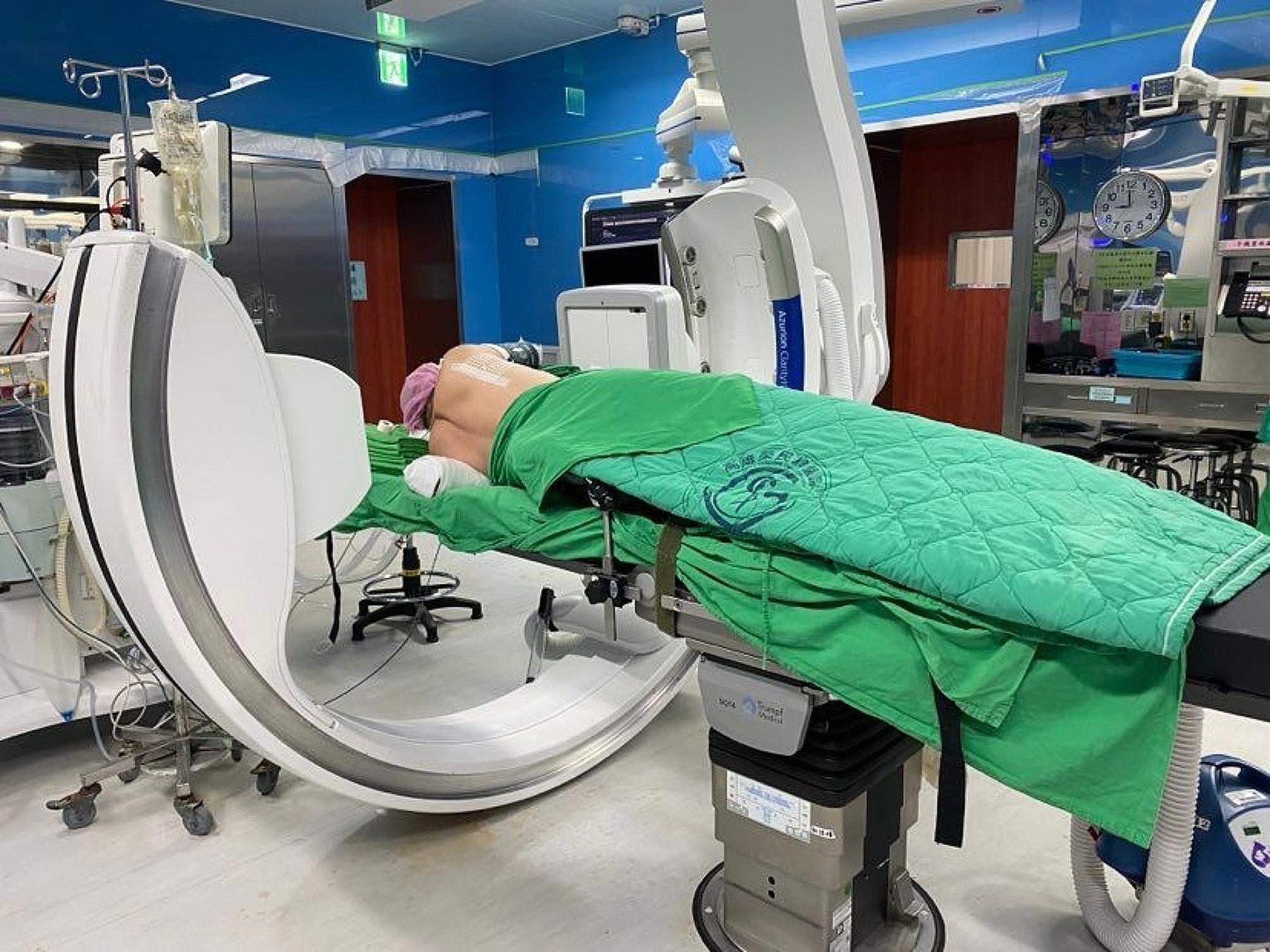
C-arm scanning patients

**Fig. 4:**
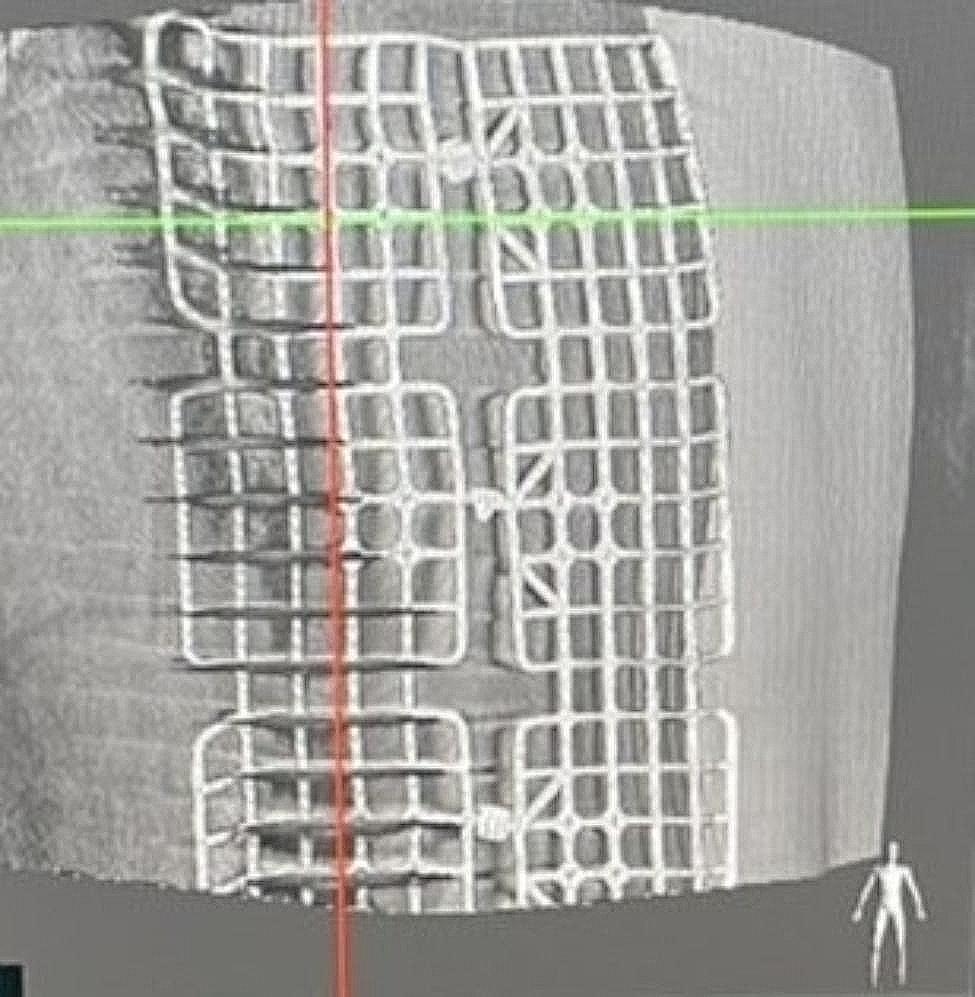
Image calculated by the program showing site for localization needle penetration

**Fig. 5:**
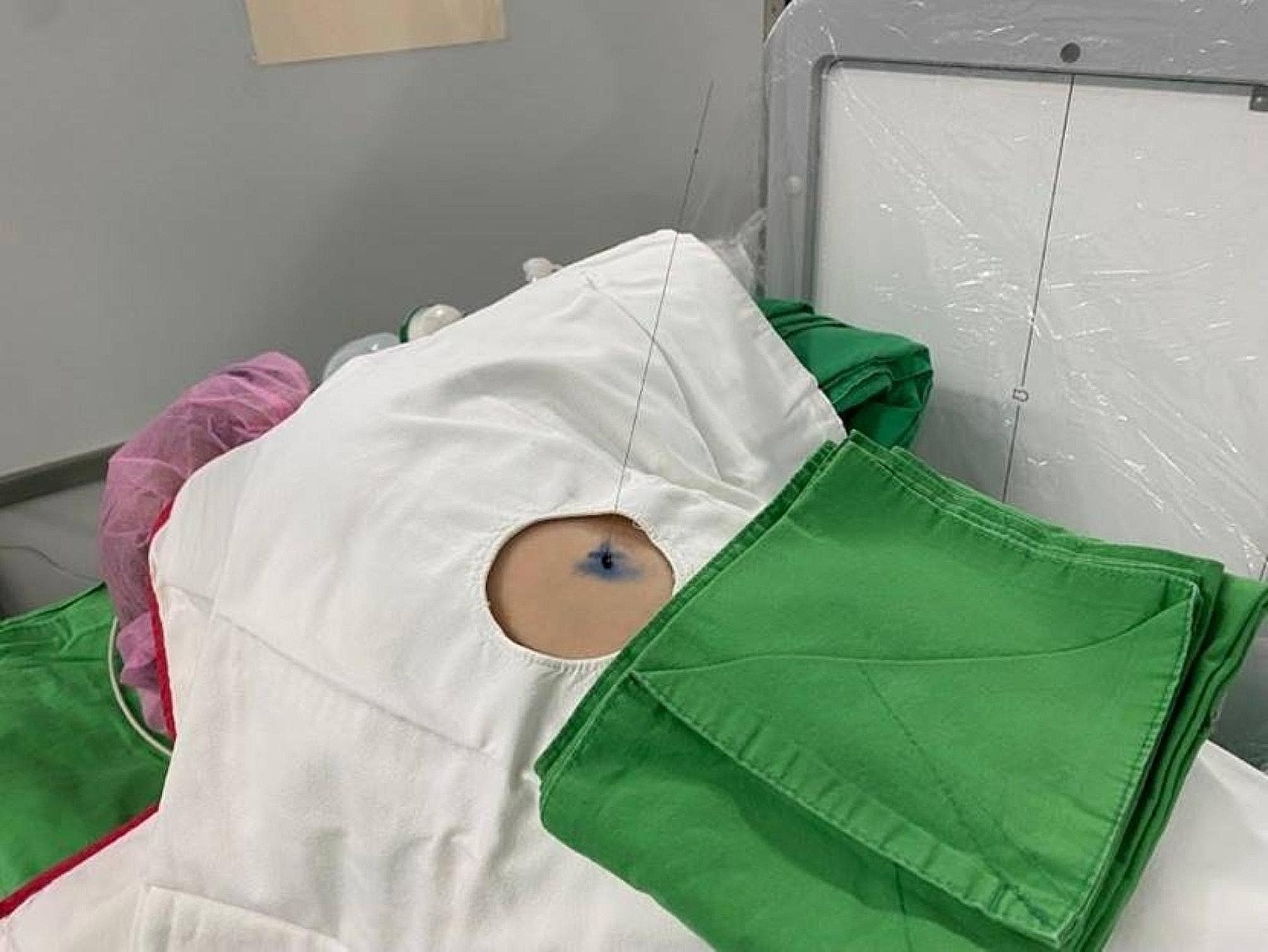
Hookwire placed into the patient’s chest wall

**Fig. 6:**
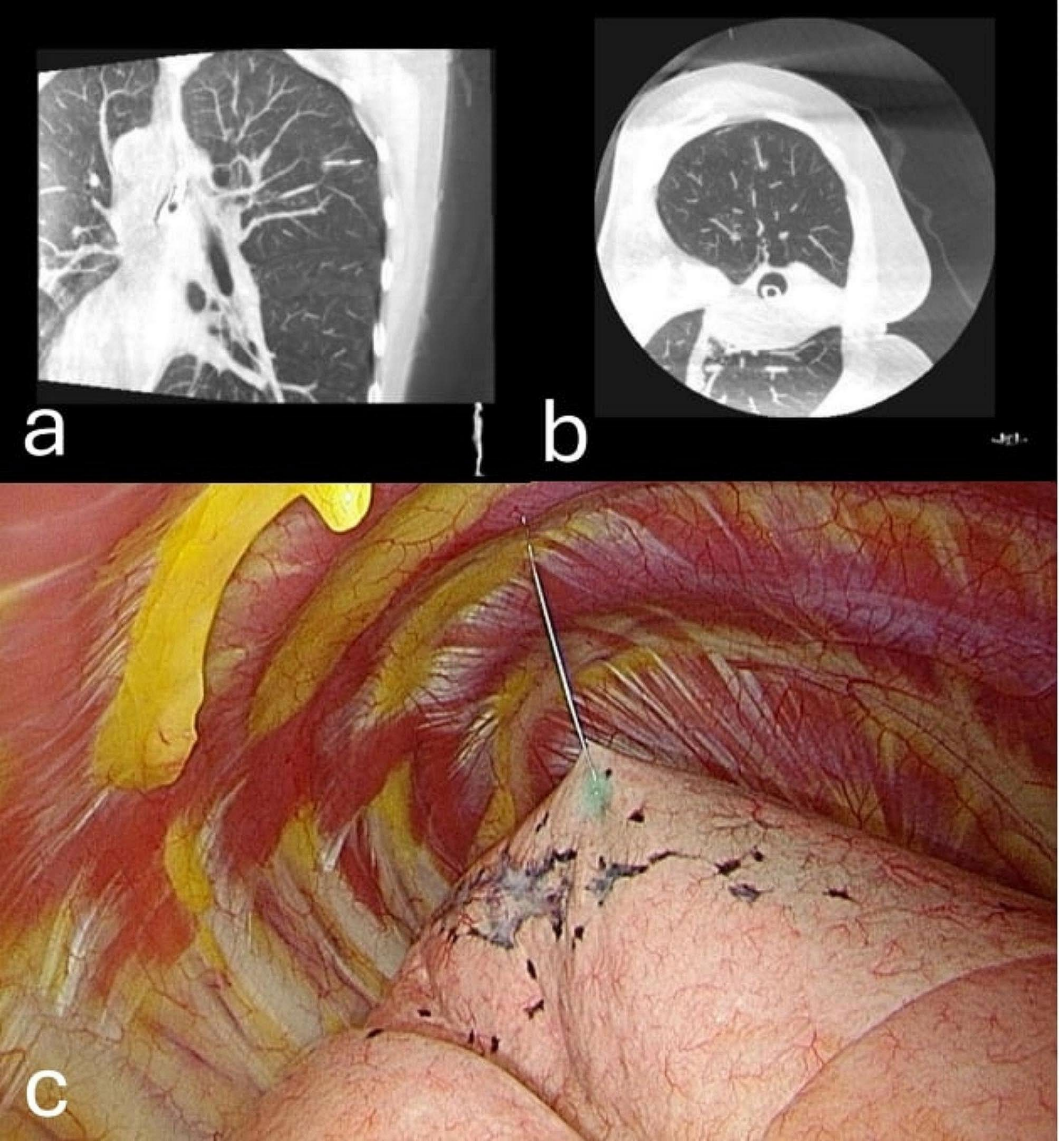
(**a**) Coronal view showing a hookwire inserted near the target lesion site. (**b**) Axial view demonstrating a hookwire inserted near the target lesion site. (**c**) Intra-operative image under thoracoscope revealing a hookwire inserted into the lung

One-stage computed tomography-guided localization was performed in the HOR equipped with a cone-beam CT apparatus (Philips Azurion system). The localization procedure and surgical resection are both performed in the same place. The localization procedure was conducted by the thoracic surgeon. First of all, the patient was sent to the HOR. Once the induction of anesthesia was completed, the patient was placed in the appropriate position for CT-guided localization. A grid was placed on the patient’s chest wall for localization (Fig. 2). Subsequently, the C-arm scanned the patient (Fig. 3), the image of the target lesion showed up on the monitor. We moved the cross to the target lesion, the software calculated the position corresponding to the position of the grid (Fig. 4). We planned the puncture site and the route of the cannula needle housing a hookwire [7]. The scanning protocol and needle route planning included the following steps. We planned the shortest route from the skin to the target lesion site. Additionally, the needle route should be avoided to be through the rib. First, the skin of the puncture site was sterilized, and one 10-cm long, 20-gauge cannula needle housing a hookwire was inserted through it. When the needle passed through intercostal space, it should be above the rib preventing from intercostal bundle injury. After establishing the plan, the needle was punctured into the soft tissue before reaching the pleura. Before inserting into the pleural, we scanned the position of needle once more. The anesthesiologist maintained two lung ventilation to achieve full lung expansion. At the same time, we inserted the needle through the pleura. Once the needle passed through the pleura, we scanned its position again. As the cannula needle approached the target site, dye was injected, and the hookwire was placed to act as a marker of the ensuring resection (Fig. 5). Finally, we scanned the position of hookwire again to ensure its proximity to the target lesion. Then, the patient stayed in the same place, and the thoracic surgery team performed video-assisted thoracic surgery (Fig. 6), usually wedge resection, for the removal of the lesion. After wedge resection was performed, the lesion would be sent for histopathological frozen section reporting. If the frozen section report revealed malignancy, a lobectomy would be performed. However, we only included the study patient undergoing wedge resection. If the study patient needed to be performed extra operation such as lobectomy, the data was excluded.

### Assessment

The primary endpoint of this study was the time for localization. Secondary endpoints were (1) time at risk during localization (2) total time under general anesthesia (3) the total time of whole intervention including localization procedure and excision of lesion (4) the occurrence of perioperative complication. The time at risk was defined as the time between the end of lesion localization and the start of skin incision.

The definition of accurate localization was that the hookwire was positioned near the lesion site. Furthermore, this localization aided the surgeon in successfully resecting the pulmonary specimen containing the lesion. The specimen was then incised to confirm whether the lesion was removed or not under inspection. In addition, the saft margin was also confirmed under inspection after examining the specimen.

### Statistics Analysis

All study data were analyzed using IBM SPSS version 26. All continuous variables are summarized as means and standard deviations and compared with Student’s t-test. Comparisons of continuous data between two groups were performed by calculating mean differences (MDs). All categorical variables are summarized as counts and percentages and compared using the χ2 test or the Fisher’s exact test. Comparisons of categorical variables between two groups were performed by calculating the percentage differences.

## Results

During the study period, 152 study patients were collected; 54 in the TSCT group and 98 in the OSCT group (Fig. 1). Two patients were excluded due to missing data. However, six patients concurrently undergoing a lobectomy and one patient concurrently under-going a segmentectomy in the TSCT group were excluded. In the other group, we also excluded three patients and two patients concurrently undergoing lobectomies and mediastinal tumor resections, respectively, in the OSCT group. Finally, 46 and 92 patients were included in the TSCT and OSCT groups, respectively.

Table 1 shows the basic characteristics of the study patients. There were significant differences in age and lesion site. The mean age of our study participants was 56.83 years. The average age of the participants in the OSCT group was 54.82 and the average age in the TSCT group is 60.23 (*p* < 0.05). There were 46 male patients (33.3%) and 92 female patients (66.7%). In the OSCT group, 26 patients (28.3%) were males and 66 patients (71.7%) were females. In the TSCT group, 20 patients (43.5%) were males and 26 patients (56.5%) were females. There were 130 patients in ASA I-II (94.3%) and eight patients in ASA III (5.7%). In the OSCT group, 86 patients (93.5%) were classified as ASA I-II and six patients (6.5%) were classified as ASA III. In the TSCT group, 44 patients were classified as ASA I–II and two patients were classified as ASA III. The proportions of right-sided lesions in the OSCT and TSCT groups were 56.5% and 78.2%, respectively (*p* < 0.05). According to previous CT findings, 128 lesions (92.8%) showed subsolid nodules (SSN) and 10 lesions (7.2%) showed solid nodules (SN). In the OSCT group, eight lesions (8.7%) were SN and 84 lesions (91.3%) were SSN. In the TSCT group, two lesions (4.3%) was SN and 44 lesions (95.7%) were SSN. The mean pulmonary lesion size on CT image finding was 7.81 mm. The mean size of pulmonary lesions was 7.86 mm and 7.74 mm in the OSCT and TSCT groups (*p* = 0.857), respectively. The distance from lesion to visceral pleura was 9.08 mm and 8.35 mm in the OSCT and TSCT groups (*p* = 0.648), respectively.

**Table 1:** Basic characteristics of the study patients

	Entire cohort	OSCT group	TSCT group	*p*-value
Number of patients	138	92	46	
Age (years)	56.83 ± 10.499	54.82 ± 10.411	60.23 ± 9.905	0.024
Sex Male Female	46 (33.3) 92 (66.7)	26 (28.3) 66 (71.7)	20 (43.5) 26 (56.5)	0.386
ASA (Amersican Society of Anesthesiologists) I–II III	130 (94.3) 8 (5.7)	86 (93.5) 6 (6.5)	44 (95.7) 2 (4.3)	0.204
Lesion site				
Right-sided Left-sided	88 (63.7) 50 (36.3)	52 (56.5) 40 (43.5)	36 (78.2) 10 (21.8)	0.023
CT findings				
Solid nodule Subsolid nodule	10 (7.2) 128 (92.8)	8 (8.7) 84 (91.3)	2 (4.3) 44 (95.7)	0.175
Lesion size on CT (mm)	7.81 ± 3.21	7.86 ± 3.45	7.74 ± 3.81	0.857
Distance from lesion to visceral pleura (mm)	8.67 ± 4.18	9.08 ± 5.81	8.35 ± 4.88	0.648

Table 2 summarizes the results of intraoperative and perioperative variables. The total number of perioperative complications of pneumothorax was six (4.3%). Only four patients (4.3%) experienced complications of pneumothorax in the OSCT group and two patients (4.3%) in the TSCT group. There was no significant difference in the incidence of perioperative complications of pneumothorax between the OSCT group and TSCT groups (*p* = 0.879). In the OSCT group, 56 patients (60.9%) were placed in the lateral decubitus position during localization and 36 patients (39.1%) were placed in the supine or prone position. In the TSCT group, 36 patients (78.2%) were placed in a supine or prone position and 10 patients (21.8%) were placed in the lateral decubitus position (*p* < 0.05). The mean blood loss was 6.93 ml in the OSCT group and 10.86 ml in the TSCT group (*p* < 0.05). After the procedure, most of the final pathological diagnoses were AIS and MIA, accounting for 90 (97.8%) and 44 (95.6%) in the OSCT and TSCT groups, respectively. The number of hook-wire dislodgement was two (2.2%) and four (8.7%) in the OSCT and TSCT groups, respectively. The localization success rates in the OSCT and TSCT groups were 97.8% and 91.3%, respectively (*p* = 0.216).

**Table 2:** Operative and perioperative variables

	Entire cohort	OSCT group	TSCT group	*p*-value
Number of patients	138	92	46	
Perioperative complications				
Pneumothorax	6 (4.3)	4 (4.3)	2 (4.3)	0.879
Position of localization				
Supine/prone Lateral decubitus	72 (52.1) 66 (47.9)	36 (39.1) 56 (60.9)	36 (78.2) 10 (21.8)	0.005
Blood loss (ml)	8.25 ± 5.19	6.93 ± 3.38	10.86 ± 7.01	0.002
Pathological diagnosis				
Benign Malignancy	4 (2.9) 134 (97.1)	2 (2.2) 90 (97.8)	2 (4.4) 44 (95.6)	0.255
Dislodgement of wire	6 (4.4)	2 (2.2)	4 (8.7)	
Localization success rate	95.6%	97.8%	91.3%	0.216

Table 3 summarizes the efficacy index variables of time. The duration of the localization procedure was significantly shorter in the OSCT group than in the TSCT group (mean difference: -15.53 min). The time at risk was also significantly lower in the OSCT group than in the TSCT group (mean difference: -145.91 min). Because the patients in the OSCT group underwent the localization procedure and surgery in the same place, the time of localization and the time at risk were much shorter. Also, there was no significant difference in the surgery duration between the OSCT group and the TSCT group (168.94 min vs. 196.10 min, *p* > 0.05). However, the time spent under general anesthesia was much longer in the OSCT group than in the TSCT group (mean difference: +64.28 min). The total time of the procedure was significantly lower in the OSCT group than in the TSCT group (mean difference: -194.93 min).

**Table 3:** Time variables

	Entire cohort	OSCT group	TSCT group	*p*-value
Number of patients	138	92	46	
Localization time (min)	21.02 ± 11.94	15.27 ± 8.18	30.80 ± 10.99	< 0.001
Time at risk (min)	88.23 ± 79.82	34.19 ± 21.25	180.10 ± 54.15	< 0.001
Operating time (min)	179.00 ± 63.08	168.94 ± 58.65	196.10 ± 67.57	0.061
Time under general anesthesia (min)	238.78 ± 74.05	262.58 ± 66.21	198.30 ± 69.84	<0.001
Total time of course (min)	276.27 ± 114.01	204.07 ± 58.80	399.00 ± 71.74	< 0.001

## Discussion

In our study, we evaluated the safety and efficacy of the OSCT and TSCT approaches to small pulmonary lesion resection. OSCT localization for small pulmonary lesions in the HOR is feasible, effective, and safe. The OSCT approach also significantly reduces the procedural time and time at risk. Therefore, patients in the OSCT group suffered from less fear and nervousness than those in the TSCT group. In our study, the mean age of the patients in the OSCT group was lower than that of patients in the TSCT group. As the use of LDCT increases, tiny pulmonary lesions are identified in more and more young patients. Therefore, this phenomenon explains why the mean age of the patients in the OSCT group is lower than that of patients in the TSCT group. The mean size of pulmonary lesions on CT images is approximately 7–8 mm, irrespective of the group in which they are found. According to NCCN guidelines, 6–8 mm pulmonary lesions in low-risk patients are preferably monitored every six months or yearly. Surgical resection is not the treatment of choice for these small lesions. In Taiwan, surgeons also explain this protocol to the patients who come to our outpatient department. However, the patients are usually nervous about the lesions in their lungs; so, they often opt for surgical resection of these lesions. If these patients prefer surgery, and we would advise them to go for the localization of the lesions for accurate surgical resection.

In our study, the average age of patients in the OSCT group (54.82) was younger than that in the TSCT group (60.23). This phenomenon potentially indicated that younger patients are more likely to receive new medical information and novel therapies. Younger patients were more likely to undergo health check-ups and low-dose CT for pulmonary examinations. The high acceptance rate of low-dose CT screening significantly resulted in a high diagnostic rate of tiny pulmonary lung lesions, which were typically pathologically proven to be early lung cancer.

In our study, the incidence of perioperative complications (such as pneumothorax) after localization and the rate of successful localization did not differ significantly between the OSCT group and the TSCT group. It indicates that localizations performed by thoracic surgeons are not inferior to those performed by an experienced radiologist. One case of pneumothorax in the OSCT group was a case of a pure ground-glass nodule over the right upper lobe. We performed localization in the HOR, and the whole procedure was smooth. However, after C-arm screening, the image showed that the localization needle was through-through from the right lower lobe to the right upper lobe with mild pneumothorax. Though the localization failed this time, we resected the target lesion successfully by tracing the pinhole on the lung’s surface to guess the location of the target lesion. One case of pneumothorax in the TSCT group was a result of hookwire dislodgement caused by severe agitation. However, we also successfully resected the target lesion by tracing the pinhole on the lung surface. Thus, localization performed by a thoracic surgeon might be better than that performed by a radiologist because the surgeon could resolve the complication in the operation room immediately if any acute situation arises.

Most patients in the OSCT group were placed in the lateral decubitus position during localization. This indicates that thoracic surgeons prefer placing patients in the lateral decubitus position because it is convenient for the surgical team to place the patient in this position to facilitate any operation that might be performed after localization. Also, most patients in the TSCT group were placed in the supine or prone position for localization. This might indicate that radiologists prefer placing patients in the supine or prone position due to the limited space available on the CT suite table. Because the CT in our hospital was not designed for intervention, it was not advisable for a radiologist to place patients in the lateral decubitus positions. The shortest possible trajectory of the localization needle was ideal for patients, and this trajectory determined the position of the patient for localization; however, the surgeon or radiologist performing the procedure could prefer specific positions for convenience.

In our study, the time of localization, time at risk, and total duration were all significantly shorter in the OSCT group than in the TSCT group. Because the patients underwent localization and surgery at the same place in the OSCT group, it saved much time that would have been used for transportation from the ward to the CT room. However, the time the patient spent under general anesthesia was significantly longer in the OSCT group than in the TSCT group, which might have increased the number of adverse events associated with anesthetics throughout the procedure. It may also have increased the cost of anesthetics, leading to an increase in the economic burden levied on patients. Extended anesthesia duration implies higher concentrations of anesthetic agents within the patient’s body. Consequently, patients may require additional time for metabolizing the anesthesia drugs. Moreover, patients are more susceptible to experiencing increased side effects of anesthesia drugs, such as postoperative nausea and vomiting (PONV).

The finding of our research could encourage more thoracic surgeons to adopt OSCT approach rather than TSCT method. However, learning curve associated with localization method should be considered when shifting to OSCT method. Thoracic surgeons could perform the localization procedure successfully and fluently when they were familiar with this new technique. Before reaching learning curve should be considered, several risks and complications during localization should also be warmed. Air embolism could happen during localization procedure. When a needle simultaneously crosses between tiny bronchus and pulmonary vein, a fistula is consequently created. The future research could find the optimal needle route to prevent from this phenomenon.

The study by Huang et al. [8], they reported a successful and safe CT-guided localization method using microcoil to localize solitary pulmonary nodules. In another study by Fang et al. [9], the technical developments were reviewed, and different localization techniques were compared. The study mentioned the advantages and disadvantages of different localization techniques. An optimal method of localization for pulmonary nodules is yet to be established [10]. Although there was a mild risk of pneumothorax and pulmonary hemorrhage due to dislodgement or migration, this technique was easier to perform and more widely used than other methods. The safety, effectiveness, and convenience of hookwire use for localization were acceptable [11, 12]. Therefore, we use the hookwire as the tool to localize the target lesion, be it during OSCT or TSCT.

In the study by Chao et al. [13], for those patients with multiple ipsilateral pulmonary nodules, OSCT localization performed in the HOR is associated with a shorter procedural time compared to the TSCT approach. In our study, we selected patients with only one pulmonary lesion undergoing wedge resection to eliminate certain confounding factors. Another radiation-free intraoperative localization approach using ultrasonography was also reported if the lung was completely deflated and could be extracted. Kondo et al. [14] reported that intraoperative ultrasonography was also a safe and effective approach to the localization of pulmonary GGN. However, we did not compare this intraoperative localization method to the OSCT method. More studies are needed to investigate different intraoperative localization methods.

Our study has several limitations. First, this was a retrospective study including a relatively small number of cases. There is some selection bias when enrolling the study patients. In addition, before our HOR was opened in 2019, we used the TSCT localization method to approach tiny pulmonary lesions. After HOR was launched in 2019, we adopted the OSCT localization approach. Some confounding factors should be considered. Thoracic surgeons might learn localization technique from radiologists’ experience during TSCT period. This possibly resulted in thoracic surgeons’ familiarity about localization technique. It could decrease procedural time when we shifted from TSCT approach to OSCT approach. Our findings should be verified by conducting a prospective study with more cases. Second, this is a single-center study. Third, we did not collect data on radiation exposure because these data were missing, and we did not calculate the cost of anesthetics. Fourth, different operators performing localization procedure have some bias in the results, various thoracic surgeons in the OSCT group and different radiologists in the TSCT group, respectively. Though these operators were all experienced in localization procedure, some confounding factors still influenced the results. In addition, our study did not compare other methods of localizing pulmonary lesions. Non-percutaneous localization approaches such as electromagnetic navigation bronchoscopy [15, 16] were not compared to the percutaneous localization method in our study. Finally, no oncological benefit could be observed in the OSCT group compared to the TSCT group because there was no significant difference in clinical outcomes between the two groups of patients with early lung cancer. We could hypothesize that less procedural time indicated less time under general anesthesia. Therefore, the side effects of anesthesia agent, such as postoperative nausea and vomiting (PONV), could be more decreased. However, we did not enroll the variables about anesthetic side effects. Significant reductions in the procedural time and the time at risk could not be further translated to clinically significant differences between the two groups.

## Conclusions

Compared to the TSCT, the OSCT-guided approach to pulmonary nodule localization in HOR significantly decreases the procedural time and risky time. The rate of successful localization and incidence of pneumothorax were similar in both groups. The OSCT-guided approach enables patients to undergo both localization and excision in a state free from anxiety. Moreover, it significantly reduces the time of potential risks, thus leading to a considerable reduction in complications related to localization. Besides, localization performed by a thoracic surgeon might be better than that performed by a radiologist because the surgeon could resolve the complication in the operation room immediately if any acute situation arises. Thus, OSCT-guided approach is the better method to localize the small lung lesions than TSCT-guided approach.

## Data Availability

The datasets used and analysed during the current study are available from the corresponding author on reasonable request.

## Abbreviations

OSCT: One-stage computed tomography
TSCT: Two-stage computed tomography
CT: Computed tomography
GGN: Ground-glass nodules
HOR: Hybrid operation room
VGHKS: Veterans General Hospital Kaohsiung
AAH: Atypical adenomatous hyperplasia
AIS: Adenocarcinoma in situ
MIA: Minimally invasive adenocarcinoma
